# Patient Identity Management in The Global Health Context

**DOI:** 10.23889/ijpds.v9i5.2494

**Published:** 2024-09-18

**Authors:** Toan Ong, Mary Pittman, Paul Biondich, Burke Mamlin, Brett Onions, Martin Suleman, Jennifer Shivers

**Affiliations:** 1 University of Colorado, Anschutz Medical Campus; 2 Regenstrief Institute; 3 Jembi Health Systems; 4 Elizabeth Glaser Pediatric AIDS foundation; 5 Regenstrief Institute

## Objective

Effective management of patient identity is crucial in population health and quality improvement. Patient identity management (PIM) in healthcare refers to the processes and technologies used to match, authenticate, and authorize patients within healthcare systems. Identifying duplicate patient records, however, is complicated by variations in how individual patients are identified within and across different systems. We aim to understand the PIM use cases, challenges, and solutions from mid and low-income countries in the Open Health Information Exchange (OpenHIE) communities to support knowledge sharing and collaboration with the goal of delivering safe and effective patient care.

## Approach

Data collection methods used at the OHIE 2023 held in Lilongwe, Malawi, from May 1st to May 5th, 2023, include:

- Cognitive collaborative hackathon: facilitation of international exchange, with participants from diverse countries elucidating the necessity for PIM within their respective nations.
- Unconferencing:  a novel and participatory approach to knowledge and information exchange prioritizes the specific interests of the attendees over a pre-established agenda.
- One-on-one interview: Country-specific discussion on PIM

## Result

In various sessions at the conference, 12 countries shared their experiences on PIM activities related to linkage methods, biometrics, data security, privacy, and policy. Key challenges include lack of connectivity, interoperability, poor data quality and compatibility, high costs, and linkages of newborn data.

## Conclusions

Low- and middle-income countries face unique challenges in developing and implementing robust PIM systems. Despite these challenges, countries in the OpenHIE community are actively working on implementing PIM systems, adopting various approaches tailored to their specific needs.

